# A Single-Copy Sensitive and Field-Deployable One-Pot RT-RPA CRISPR/Cas12a Assay for the Specific Visual Detection of the Nipah Virus

**DOI:** 10.1155/2024/4118007

**Published:** 2024-11-20

**Authors:** Kaikai Jin, Pei Huang, Boyi Li, Zengguo Cao, Zanheng Huang, Zimo Zhang, Meihui Liu, Hao Li, Lijuan Niu, Tianyi Zhang, Yuanyuan Li, Xuemeng Li, Hualei Wang, Haili Zhang

**Affiliations:** ^1^State Key Laboratory for Diagnosis and Treatment of Severe Zoonotic Infectious Diseases, Key Laboratory for Zoonosis Research of the Ministry of Education, Institute of Zoonosis, College of Veterinary Medicine, Jilin University, Jilin 130062, China; ^2^Key Laboratory of Special Pathogens and Biosafety, Wuhan Institute of Virology, Center for Biosafety Mega-Science, Chinese Academy of Sciences, Hubei 430071, China; ^3^Guangdong Provincial Key Laboratory of Medical Molecular Diagnostics, School of Basic Medicine, Guangdong Medical University, Dongguan, Guangdong 523000, China; ^4^The Marine Biomedical Research Institute, Affiliated Hospital of Guangdong Medical University, Guangdong Medical University, Zhanjiang, Guangdong 524023, China

**Keywords:** CRISPR/Cas12a system, nipah virus, one-pot, reverse transcription-recombinase polymerase amplification (RT-RPA), viral nucleic acid detection

## Abstract

Nipah virus (NiV) is an emerging bat-borne zoonotic virus that can be transmitted to humans and other animals through infected bats or contaminated foods. The disease is highly lethal in humans (40%–75%) and has the potential for human-to-human transmission. Currently, there are no approved treatments or vaccines for NiV infection in humans or animals. Consequently, there is a pressing need for a highly sensitive, precise, and visually detectable assay to enable early intervention and mitigate the transmission of NiV infection. Here, we report a single-copy sensitive, field-deployable, one-pot visual reverse transcription-recombinase polymerase amplification (RT-RPA)-clustered regularly interspaced short palindromic repeat (CRISPR)/CRISPR associate system (Cas)12 for the detection of NiV. The assay works by targeting the *N* gene of NiV, and the results are directly visible to the naked eye. The assay has demonstrated the ability to detect as few as 5.5 copies/μl of positive plasmids or 5.5 × 10^1^ copies/μl of RNA transcripts when reacted at constant temperature for 40 min. It showed high specificity for NiV and had no cross-reaction with other pathogens, including rabies virus (RABV), Japanese encephalitis virus (JEV), herpes simplex virus type 1 (HSV-1), Hendra virus (HeV), and *Streptococcus suis* (*S. suis*), that can cause clinical symptoms similar to those of NiV infection. Moreover, this assay had a 100% coincidence rate with the reverse transcription quantitative polymerase chain reaction (RT-qPCR) method recommended by the World Organization for Animal Health (WOAH) for the detection of simulated clinical samples, indicating that it has great potential as an ultrasensitive, simple, and portable novel assay for the onsite diagnosis of NiV infection.

## 1. Introduction

Nipah virus (NiV) is a novel zoonotic virus belonging to the subfamily Paramyxovirinae of the Paramyxoviridae family in the genus *Henipavirus* [1]. NiV was first discovered in domestic pigs in Malaysia and Singapore in 1998 and 1999 [2], after which more than 1 million pigs were destroyed to control its spread. Fruit bats have been found to be the primary carriers of NiV and have played a role in NiV outbreaks across various regions worldwide [3]. NiV infects not only humans and pigs but also other mammals, such as goats, dogs, cats, and horses [4]. The main ways by which people and other animals can be infected with NiV are by drinking date palm juice tainted by NiV-infected fruit bats [5] or by direct contact with diseased animals (e.g., infected pigs or bats) or their body fluids (e.g., saliva, urine, or blood) [6].

Additionally, there is a possibility of human-to-human transmission. Infection with NiV can lead to various clinical symptoms in both humans and animals, ranging from asymptomatic cases (subclinical infections) to severe respiratory infections and potentially fatal encephalitis [7]. Numerous recurrent outbreaks have been documented in South Asia and Southeast Asia in recent years [8, 9], including new cases reported in India and Bangladesh during 2023. These new outbreaks have caused 14 cases, and 7 people have died [10, 11]. Thus, NiV not only has a severe destructive effect on the healthy development of the farming industry but also seriously endangers human health and safety. To date, no vaccine is available, and there is no treatment for either people or animals following infection with NiV. Therefore, early detection of NiV infection remains the best method for controlling NiV outbreaks.

Numerous laboratory diagnostic procedures have been established for the detection of NiV. To date, virus isolation is the simplest and most reliable assay for diagnosing NiV infection, and the serum neutralization test (SNT) is the gold standard; however, both assays need to be performed in a biosafety level 4 (BSL-4) laboratory, which greatly limits the applicability and popularity of these methods [12, 13]. Enzyme-linked immunosorbent assay (ELISA) is a relatively simple procedure that can be used for high-throughput screening (HTS) of suspected NiV samples. Owing to its high immunogenicity, the NiV N protein is often used as an antigen in ELISA. Several indirect ELISA methods for detecting recombinant NiV N protein have been developed for porcine and human serum samples [14, 15]. In recent years, the development of pathogen nucleic acid tests, which are important for the early detection of infections with high sensitivity and specificity, has occurred [16]. As early as 2000, the US Centers for Disease Control and Prevention (CDC) designed an reverse transcription polymerase chain reaction (RT-PCR) method to detect the *N* gene of NiV. A duplex nested RT-PCR method with an internal control (IC) was subsequently developed, which further improved the reliability of the RT-PCR method for detecting NiV [17]. Several real-time RT-quantitative PCR (RT-qPCR) assays have also been established for detecting NiV [18], and this method has been defined as the gold standard for the clinical detection of NiV infection by the World Health Organization (WHO) [19]. qPCR is more sensitive, specific, and accurate in quantifying viral concentrations than traditional PCR assays are [20]; however, this method requires qualified laboratory workers and expensive equipment. Because epidemics of NiV frequently occur in regions with limited resources, there is a pressing need to establish a sensitive, quick, point-of-care diagnostic technique that does not require the use of precision equipment. One potentially useful technique is the clustered regularly interspaced short palindromic repeats (CRISPRs)/CRISPR associate system (Cas) system.

In contrast to the assays mentioned above, the CRISPR/Cas system stands out because of its greater specificity, usability, and portable diagnostic features [21]. On the basis of the associated functionality of Cas proteins [22], a range of sophisticated CRISPR-based diagnostics (CRISPR-Dx) have been developed, including SHERLOCK [23], DETECTR [24], and HOLMES [25]. These techniques satisfy the need for point-of-care testing tools that are sensitive, rapid, and easy. CRISPR-Cas–based nucleic acid assays usually require recombinase polymerase amplification (RPA) and PCR for nucleic acid preamplification [22]. However, traditional CRISPR-Dx performs separate nucleic acid preamplification, leading to an elevated level of complexity in the assay procedure and potentially increasing the likelihood of residual contamination caused by the transfer of amplification products.

Therefore, in this study, we combined CRISPR/Cas12a with reverse transcription-RPA (RT-RPA) and enclosed the reactions in an airtight environment. We thus established a one-pot visual RT-RPA-CRISPR/Cas12a rapid detection assay for NiV RNA. The assay is ultrasensitive and portable and is expected to provide technical support for early and rapid diagnosis in remote localities such as farms where outbreaks of NiV occur.

## 2. Materials and Methods

### 2.1. Nucleic Acids From Pathogens, RNA Transcripts, and Plasmids

Nucleic acids originating from herpes simplex virus type 1 (HSV-1), *Streptococcus suis* (*S. suis*), and rabies virus (RABV) were purified via DNA/RNA extraction kits (BioPerfectus China). The viral RNAs of NiV, Japanese encephalitis virus (JEV), and the closely related paramyxovirus Hendra virus (HeV) were stored in a BSL-4 laboratory at the Wuhan Institute of Virology (WIV). RNA transcripts of the NiV *N* gene fragment (GenBank: NC_002728) were synthesized in vitro by Sangon Biotech, China. The recombinant plasmid pcDNA3.1-NiV-N was created by incorporating the entire length of the *N* gene (GenBank: NC_002728) into the vector pcDNA3.1.

### 2.2. Design of Primers, Probes, and CRISPR RNAs (crRNAs)

A total of 112 NiV *N* gene sequences were obtained from the GenBank database (https://www.ncbi.nlm.nih.gov/) and aligned via multiple alignment using fast fourier transform (MAFFT) version 7. The highly conserved regions of the *N* gene were identified as targets for designing specific RPA primers via Prime Primer 5.0 software. Subsequently, online tools (http://www.rgenome.net/) were utilized to design specific crRNAs. The designed primers and probes were synthesized by Sangon Biotech, China. All sequences used in this study are given in Table 1.

### 2.3. crRNA Preparation

The crRNAs were prepared according to a previous study, with slight modifications [24]. First, double-stranded DNA (dsDNA) with a T7 promoter was prepared as follows: The non-target strand (NTS), comprising a T7 promoter, an Lachnospiraceae bacterium Cas12a (LbCas12a) scaffold sequence, and target sequences, was mixed with the target strand (TS), which is the complementary strand of the LbCas12a scaffold sequence and target sequences, in a hybridization buffer (20 mM Tris-HCl, pH 7.5; 100 mM KCl; 5 mM MgCl_2_). The mixture was incubated at 95°C for 5 min. Subsequently, the temperature was reduced slowly to room temperature [24]. The dsDNA template was subsequently incubated with T7 polymerase at 37°C for a period of 12–16 h, utilizing the HiScribe T7 High Yield RNA Synthesis Kit (New England Biolabs (NEB), Beijing, China), with the objective of obtaining crRNA. The crRNAs were purified via the Monarch RNA Cleanup Kit (NEB, Beijing, China), and the concentrations of the crRNAs were determined via an Implen N60 ultraviolet–visible (UV–Vis) Spectrophotometer.

### 2.4. Establishment and Optimization of the CRISPR/Cas12a Cleavage System

The LbCas12a protein was purified and stored in our laboratory at a concentration of 2.72 mg/ml. The crRNAs were first screened in a 20 μl CRISPR/Cas12a cleavage system, which contained 1250 nM Cas12a protein, 100 nM crRNA, 500 nM 5′-carboxyfluorescein (FAM)-TTATT-black hole quencher (BHQ)-3′ probe, 100 ng pcDNA3.1-NiV-N, and 2 μl of 10 × Borealis buffer. Different concentrations of the Cas12a protein (750, 1000, 1250, 1500, and 1750 nM) were then added separately to the CRISPR/Cas12a reaction system to determine the optimal concentration of the Cas12a protein. After the optimal protein concentration was determined, the crRNA concentration was adjusted to 50, 75, 100, 125, or 150 nM to screen for the optimal concentration of crRNA. After that, the buffers commonly used for Cas12a, including NEB1.1, NEB2.1, NEB3.1, CutSmart (NEB) and Borealis buffer, were screened. The nontargeting groups were used as negative controls. The samples were incubated at 37°C for 40 min in a quantitative fluorescence PCR instrument (StepOnePlus Real-Time Fluorescence PCR System), and fluorescence signals were collected at 1-min intervals.

### 2.5. Establishment and Optimization of a One-Pot Visual RT-RPA-CRISPR/Cas12 Assay

The RT-RPA reaction was carried out via a TwistAmp R Basic kit (TwistDx, UK) according to the manufacturer's instructions. The RPA powders were diluted with 29.5 µl of rehydration buffer, 2.5 µl of magnesium acetate, 2.4 µl of forward primer/reverse primer (F/R; 10 µM), and 2 U/µl Superscript IV reverse transcriptase (Thermo Fisher Scientific, Baltics UAB). DEPC-treated water was added to a final volume of 45 µl. Then, 20 µl of the premixed solution was transferred to another tube, and 5 µl of the target was added for the RT-RPA reaction. The components of the CRISPR/Cas12a reaction system were scaled up 2.5-fold at their optimal ratio to ensure that their final concentrations in the 50 µl Cas12a reaction were optimal after mixing with the RT-RPA reaction mixture.

Three amplification temperatures (37, 39, and 42°C) and three reaction times (10, 20, and 30 min) for RT-RPA were screened separately, and different concentrations of positive plasmids were used. All of the experiments described above were carried out three times. The results were observed with the naked eye or recorded on a smartphone. The RGB (red, green, and blue channels of colour) pixel values were extracted from the images via ImageJ, and finally, the recognition results were displayed.

### 2.6. Evaluating the Sensitivity and Specificity of the One-Pot Visual RT-RPA-CRISPR/Cas12 Assay

Tenfold serial dilutions (10^10^–10^−1^ copies/μl) were made of the pcDNA3.1-NiV-N plasmids and the RNA transcripts separately. Different concentrations of plasmids or RNA transcripts were then used as templates to evaluate the sensitivity of the one-pot assay. Nucleic acids from other pathogens, including RABV, JEV, HSV-1, HeV, and *S. suis*, which can cause neurological symptoms similar to those of NiV, were also tested via a one-pot assay to evaluate the specificity of this assay.

### 2.7. Simulated Clinical Sample Preparation and RT-qPCR Assay

The total RNA from Vero E6 cells infected with NiV was extracted and purified in the BSL-4 laboratory at WIV. The purified RNA was then mixed with the RNA extracted from healthy human saliva samples to simulate NiV clinical infection. Among the 60 samples tested, 27 samples were healthy human RNA samples spiked with NiV RNA and 33 samples were healthy human RNA samples without NiV RNA. These samples were assessed via both a one-pot assay and an RT-qPCR assay. The RT-PCR assay is the standard test recommended by the World Organization for Animal Health (WOAH) (https://www.woah.org/fileadmin/Home/eng/Health_standards/tahm/3.01.15_NIPAH_HENDRA). The primers used for RT-qPCR are shown in Table 1.

### 2.8. Statistical Analysis

To compare the groups, one-way analysis of variance (ANOVA) was employed, and the subsequent statistical analyses were conducted via GraphPad Prism. The data are presented as the means ± standard errors. *p* values less than 0.05 indicate statistically significant differences, which are denoted by the following symbols: *⁣*^*∗*^*P* ≤ 0.05; *⁣*^*∗∗*^*P* ≤ 0.01; *⁣*^*∗∗∗*^*P* ≤ 0.001; *⁣*^*∗∗∗∗*^*P* ≤ 0.0001.

## 3. Results

### 3.1. Scheme of the One-Pot Visual RT-RPA-CRISPR/Cas12 Assay

**Figure 1:**
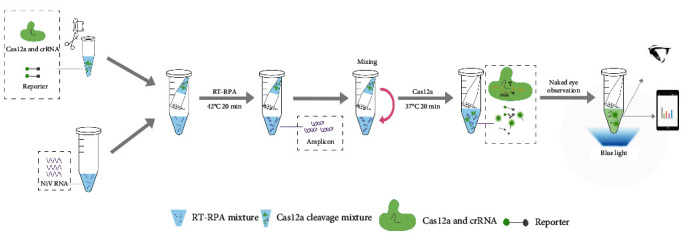
Schematic illustration of the one-pot visual RT-RPA-CRISPR/Cas12 assay for the detection of NiV infection. Cas, CRISPR associate system; CRISPR, clustered regularly interspaced short palindromic repeat; NiV, nipah virus; RT-RPA, reverse transcription-recombinase polymerase amplification.

The RT-RPA reaction mixtures containing clinical samples were added to the bottom of an eppendorf (EP) tube. The CRISPR/Cas12a reaction system was added to the bottom of a PCR tube, which was then inverted into the EP tube containing the RT-RPA reaction. Following the sealing of the EP tubes, the targets were amplified in a thermal cycler or water bath maintained at 42°C for a 20-min period. The tubes were subsequently and instantaneously centrifuged to mix the CRISPR/Cas12a reaction system and RT-RPA reaction components, followed by a 20-min reaction period at 37°C. When the samples were NiV positive, the crRNA-induced binding of the amplification products (dsDNA) to the Cas12a protein resulted in cleavage of the single-stranded DNA fluorophore-quencher (ssDNA-FQ) reporter probe, which then produced green fluorescence. The results were observed by mobile phone or the naked eye with a blue light meter (Major Science, Shanghai; Figure 1).

### 3.2. Screening of NiV N-Specific crRNAs

**Figure 2:**
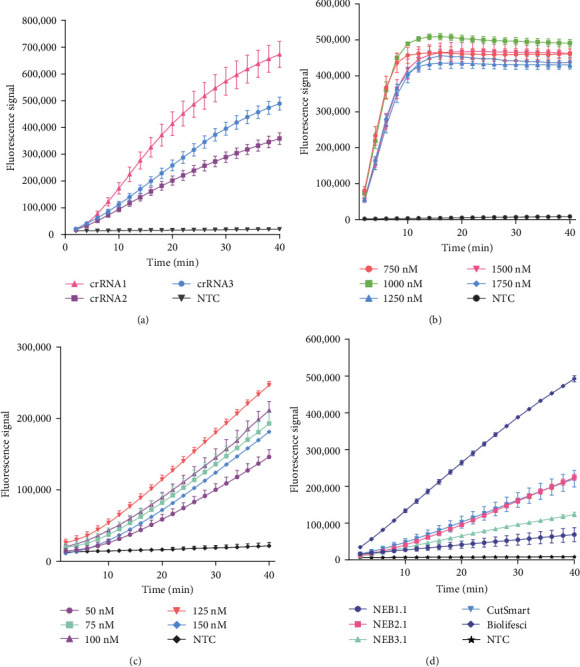
Optimization of the CRISPR/Cas12a system. (a) Three crRNAs were screened against 100 ng of NiV *N*-positive plasmid in a total reaction volume of 20 μl. The fluorescence intensities were monitored via real-time PCR. (b) A one-pot assay was then performed using different concentrations of Cas12a proteins, with the concentration of crRNA fixed at 100 nM. (c) A one-pot assay was performed using different concentrations of crRNA, but the concentration of the Cas12a protein was fixed at 1000 nM. (d) Four different CRISPR/Cas12a reaction buffers were evaluated. The error bars represent the means ± s.d. from three replicates (*n* = 3). Cas, CRISPR associate system; CRISPR, clustered regularly interspaced short palindromic repeat; NiV, nipah virus; NTC, nontarget control reaction; PCR, polymerase chain reaction; s.d., standard deviation.

Three crRNAs targeting the *N* gene were designed, of which crRNA1 and crRNA2 matched exactly with the 112 NiV *N* gene sequences, and crRNA3 matched exactly with 89 out of 112 NiV *N* gene sequences (Figure S1a, b). We screened the crRNAs to achieve high detection sensitivity. Three of the crRNAs were able to trigger Cas12a to cleave the ssDNA-FQ reporter when the positive plasmids were detected. The crRNA with the strongest ability to activate the Cas12a protein was crRNA1, so it was chosen for subsequent experiments (Figure 2a).

### 3.3. Optimization of the CRISPR/Cas12a System

The concentration of the Cas12a protein in the CRISPR/Cas12a reaction system was optimized by adjusting its concentration to 750, 1000, 1250, 1500, or 1750 nM. The results revealed that the trans-cleavage activity of the Cas12a protein was highest when the concentration was 1000 nM (Figure 2b). Next, the concentration of crRNA in the reaction system was optimized, and the results suggested that the trans-cleavage activity of CRISPR/Cas12a was optimal when the concentration of crRNA was 125 nM (Figure 2c). Finally, four different commercial CRISPR/Cas12a reaction buffers were evaluated. The results demonstrated that the various buffers had notable effects on the cleavage activity of CRISPR/Cas12a. Among these, Cas12a exhibited the most pronounced trans-cleavage activity in the Biolifesci Lachnospiraceae bacterium Cpf1 (LbCpf1) buffer (Figure 2d). These findings suggest that the CRISPR/Cas12a reaction system is most effective when the reaction contains 1000 nM Cas12a protein and 125 nM crRNA and occurs in Biolifesci LbCpf1 buffer.

### 3.4. Optimization of the One-Pot Visual RT-RPA-CRISPR/Cas12 Assay

**Figure 3:**
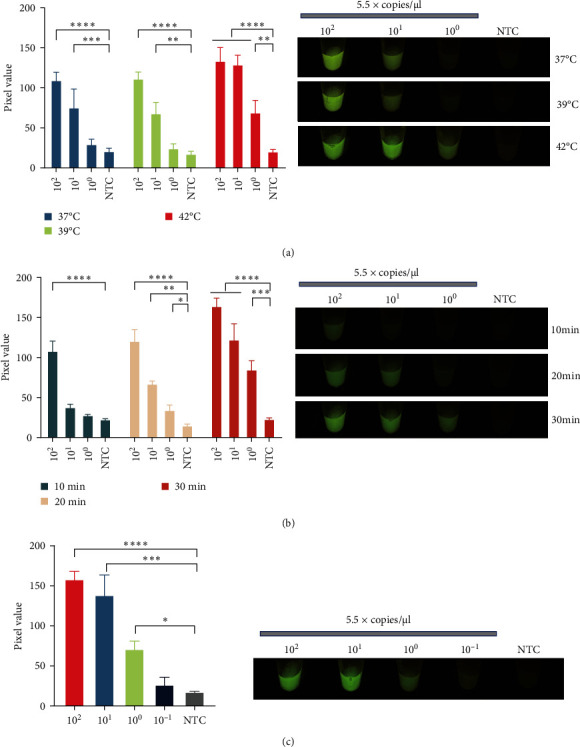
Optimization of the one-pot visual RT-RPA-CRISPR/Cas12 assay. (a) The optimal temperature of the one-pot assay was explored by assessing tenfold serial dilutions of the pcDNA3.1-NiV-N plasmid (5.5 × 10^2^−10^−1^ copies/μl). (b) The optimal reaction time of the one-pot assay was tested by assessing tenfold serial dilutions of the pcDNA3.1-NiV-N plasmid (5.5 × 10^2^−10^−1^ copies/μl). (c) The sensitivity of the one-pot assay was evaluated by using a tenfold serial dilution of the pcDNA3.1-NiV-N plasmid (5.5 × 10^2^−10^−1^ copies/μl) as a template. The pixel intensities of the green light channel were collected and analyzed from images taken with a smartphone (a–c). The error bars represent the means ± s.d. of three replicates (*n* = 3). *⁣*^*∗*^*P* ≤ 0.05; *⁣*^*∗∗*^*P* ≤ 0.01; *⁣*^*∗∗∗*^*P* ≤ 0.001; *⁣*^*∗∗∗∗*^*P* ≤ 0.0001. Cas, CRISPR associate system; CRISPR, clustered regularly interspaced short palindromic repeat; NiV, nipah virus; NTC, nontarget control reaction; RT-RPA, reverse transcription-recombinase polymerase amplification; s.d., standard deviation.

To further improve the sensitivity of the one-pot assay, the RT-RPA reaction conditions were optimized. The optimal temperature for an RT-RPA amplification reaction is usually 37–42°C. The optimization of the reaction temperature demonstrated that a single copy of the target could be detected in a one-pot assay conducted at 42°C within 40 min (Figure 3a). After that, the impact of the RT-RPA amplification time on the sensitivity of the one-pot assay was investigated. The results showed that the fluorescence signal of the one-pot assay increased with increasing RT-RPA amplification time (Figure 3b). Because the assay designed here should be rapid, a 20-min RT-RPA amplification time was selected for the subsequent experiments. Next, the sensitivity of the one-pot assay was investigated by examining its ability to detect tenfold serial dilutions of pcDNA3.1-NiV-N plasmids (5.5 × 10^2^−10^−1^ copies/μl). The results revealed that the detection limit of the one-pot assay was 5.5 copies/μl (Figure 3c).

### 3.5. Sensitivity and Specificity Evaluation of the One-Pot Visual RT-RPA-CRISPR/Cas12 Assay

**Figure 4:**
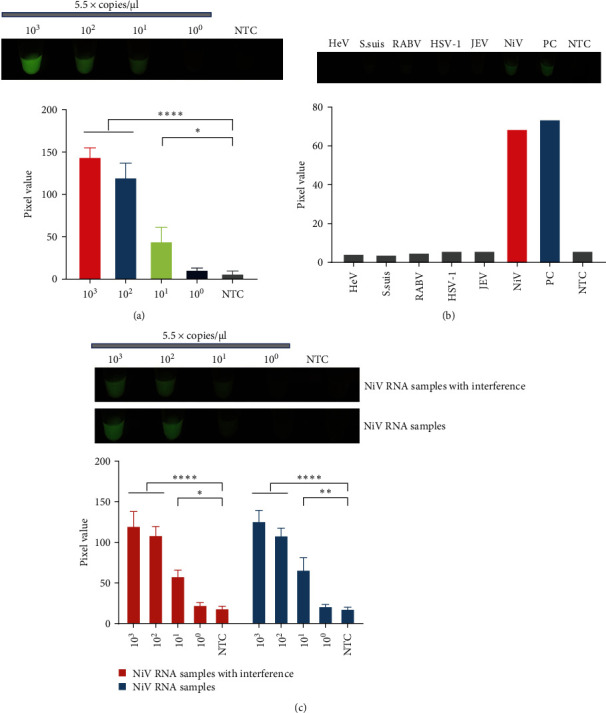
Sensitivity and specificity evaluation of the one-pot visual RT-RPA-CRISPR/Cas12 assay. (a) The sensitivity of the one-pot assay was evaluated via the use of a tenfold serial dilution of synthetic NiV *N* RNA transcripts as templates. (b) The specificity of the one-pot assay for the detection of NiV. (c) Specificity and stability of the one-pot assay in the detection of simulated complex clinical samples. The pixel intensities of the green light channel were collected and analyzed from images taken with a smartphone (a–c). The error bars represent the means ± s.d. of three replicates (*n* = 3). *⁣*^*∗*^*P* ≤ 0.05; *⁣*^*∗∗*^*P* ≤ 0.01; *⁣*^*∗∗∗∗*^*P* ≤ 0.0001. Cas, CRISPR associate system; CRISPR, clustered regularly interspaced short palindromic repeat; HeV, Hendra virus; HSV-1, herpes simplex virus type 1; JEV, Japanese encephalitis virus; NiV, nipah virus; NTC: nontarget control reaction; PC, NiV positive plasmid control; RABV, rabies virus; RT-RPA, reverse transcription-recombinase polymerase amplification; s.d., standard deviation; *Streptococcus suis* (*S. suis*).

To evaluate the sensitivity of our established one-pot assay, tenfold serial dilutions of NiV RNA transcripts were prepared and used as the detection templates. The results revealed that the detection limit of the one-pot assay for NiV RNA transcripts was 55 copies/μl (Figure 4a). The nucleic acids from RABV, JEV, HSV-1, HeV, and *S. suis*, which can cause clinical symptoms similar to those observed in NiV infection, were subsequently used to assess the specificity of the one-pot assay. The results revealed that only the pcDNA3.1-NiV-N plasmid and NiV RNA groups produced high fluorescence signals, whereas the other groups and the negative control group produced no fluorescence signals (Figure 4b). The NiV RNAs were then diluted with a mixture of nucleic acids from RABV, JEV, HSV-1, HeV, and *S. suis*. The one-pot assay showed a similar sensitivity when detecting this mixture to that of the pure NiV RNA samples, which was as low as 55 copies/μl. This finding indicates that this one-pot assay has good specificity and stability in detecting both simple and clinically complicated infection samples (Figure 4c).

Moreover, the feasibility of conducting the assay reactions at room temperature (23−25°C) was explored. The results showed that the assay could detect 5.5 × 10^3^ copies/μl of positive plasmids within 1 h at room temperature, and the same results could be obtained in a nonlaboratory environment (Figure S2). Although the sensitivity was lower than that at higher temperatures, being able to conduct the assay at room temperature eliminates the need for instruments, meaning that the one-pot assay could be adapted to extreme detection environments.

### 3.6. Evaluation of the One-Pot Visual RT-RPA-CRISPR/Cas12a Assay in the Detection of NiV RNA Spiked Into Human RNA Samples

**Figure 5:**
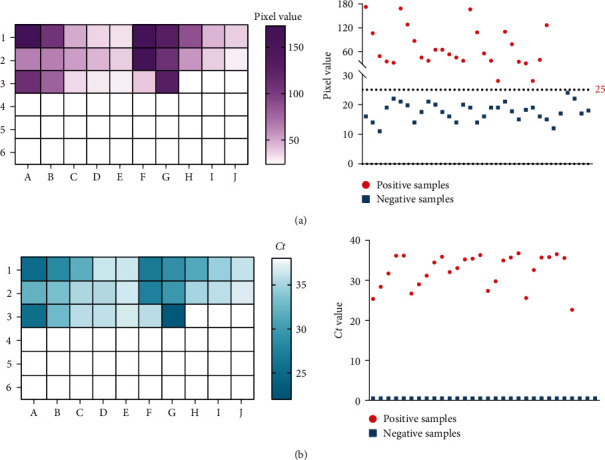
Evaluation of the one-pot visual RT-RPA-CRISPR/Cas12a assay in the detection of NiV RNA spiked into human RNA samples. (a) The simulated clinical samples (27 positive samples and 33 negative samples) were tested via a one-pot assay. The pixel intensities of the green light channel were collected and analyzed from images taken with a smartphone. Pixel intensity values above 24 were considered positive results. (b) The simulated clinical samples (27 positive samples and 33 negative samples) were assessed via RT-qPCR. A *Ct* value ≤40 was considered a positive result. Cas, CRISPR associate system; CRISPR, clustered regularly interspaced short palindromic repeat; NiV, nipah virus; RT-RPA, reverse transcription-recombinase polymerase amplification.

To demonstrate the applicability of the assay in clinical samples, simulated clinical samples were prepared by mixing different concentrations of RNA from NiV-infected cells with RNA from human saliva. These experiments were conducted in a BSL-4 laboratory, and 27 positive and 33 negative samples were finally prepared. The simulated clinical samples were then assessed via the one-pot assay developed here, as well as the RT-qPCR recommended by WOAH. The one-pot assay was able to distinguish 27 positive samples from 33 negative samples effectively (Figure 5a). RT-qPCR was also able to identify 27 positive samples, with *Ct* values ranging from 24–39, and 33 negative samples, which had no cycle threshold (*Ct*) value (Figure 5b). The assay developed here, therefore, had a 100% coincidence rate with RT-qPCR and can be used for the detection of NiV in clinical samples.

## 4. Discussion

NiV infection is highly lethal in humans (40%–75%) [2], is zoonotic, and can be transmitted from person to person, making the WHO [19] classify it as a priority pathogen on their research and development (R&D) blueprint list, signifying an imperative requirement for accelerated research and development initiatives concentrated on NiV. Given the absence of a vaccine or therapeutic agent approved for use in humans and other animals [26], the early identification of NiV through diagnostic techniques is essential for the timely treatment of patients and the prevention of outbreaks.

Although several rapid tests are available for the detection of NiV, these tests have a few shortcomings. An isothermal reverse transcription-loop-mediated isothermal amplification (RT-LAMP) assay targeting the NiV *N* gene was recently reported, with a detection limit of approximately100 pg of NiV pseudovirus RNA at 65°C [27]. RT-LAMP, however, requires a high reaction temperature (65°C) and complex oligonucleotide design. The RT-RPA technique, another isothermal amplification technique, has potential advantages over other techniques, such as RT-LAMP. It can be performed at near-ambient temperatures (37−42°C) and does not necessitate the intricate design of oligonucleotides. However, RPA tolerates mismatches of up to 9 bases [28, 29], increasing the risk of amplifying nontarget sequences and resulting in false positives [30–32]. The CRISPR/Cas systems are capable of distinguishing targets with a single base pair difference with high specificity, and they have been widely used in nucleic acid detection strategies owing to their specific collateral cleavage activity [25, 33]. As the RPA reaction takes place at a constant temperature (30−42°C, preferably near 37°C) [34] and the Cas12a protein can specifically target DNA at 37°C, we chose Cas12a here to adapt better to the reaction temperature of RPA. Moreover, to solve the problem of aerosol contamination after RPA amplification, we put the RT-RPA and CRISPR/Cas 12a reactions in a single tube to avoid opening the lids and minimize potential cross-contamination.

NiV infections have been reported in Malaysia, Singapore, Bangladesh, and India, and there has also been a suspected outbreak in the Philippines [35]. The strains transmitted in India and Bangladesh are different from the strains present in Malaysia [11]. To detect all currently known NiV strains, we compared 112 NiV *N* gene sequences, including NiV-B (transmitted in Bangladesh and India), NiV-M (strains transmitted in Malaysia), and NiV-T (transmitted in Thailand), to search for shared sequence targets. The conserved sequences on the NiV *N* gene were then selected and used as assay targets for RPA primer screening and crRNA design. We found that crRNA1 was an exact match to the target sequences from all 112 strains and was able to produce the best cleavage activity with the CRISPR/Cas12a system. By optimizing the reaction conditions, the sensitivity of the one-pot assay established in this study reached 5.5 copies/μl for positive plasmids and 55 copies/μl for NiV viral RNA when reacted at a constant temperature for 40 min. We speculated that the decreased sensitivity of the assay for NiV viral RNA may be due to the loss of RNA during the reverse transcription process.

In addition, this assay had excellent specificity and sensitivity to NiV, and the presence of other pathogens that can cause similar symptoms did not interfere with the results (Figure 4), indicating that this assay has the potential to be used in NiV-infected clinical samples. To ensure the reliability and specificity of the one-pot assay in assessing real clinical samples, we simulated clinical samples by mixing RNAs from NiV-infected cells with RNAs from human saliva in a BSL-4 laboratory. The results showed that our assay was able to identify 27 positive samples and 33 negative samples effectively, showing a 100% coincidence rate with RT-qPCR (Figure 5). Moreover, this assay requires minimal equipment or staff, making it suitable for rapid testing on farms and at the point of care. Although the thermostatic device we used for the reactions was portable, extreme conditions may be encountered when the assays are performed in the field, so we tested the efficacy of the one-pot assay in a room temperature environment. We found that the assay was able to detect 5.5 × 10^3^ copies/μl of positive plasmids at room temperature, even in a nonlaboratory setting (Figure S2), demonstrating that it has the potential to be used in extreme environments despite the sacrifice in sensitivity. Future work will focus on the optimization of the reaction to allow clinical detection of viral RNA at room temperature.

## 5. Conclusion

In conclusion, we present a one-pot assay for visualizing NiV by combining RPA amplification and Cas12a cleavage. The detection sensitivity is single-copy, and the whole detection process can be completed within 40 min. The proposed assay requires minimal equipment and only very simple steps to complete, providing a new ultrasensitive, simple, and portable assay for onsite diagnosis to allow NiV disease detection and preventive surveillance in resource-poor areas.

## Figures and Tables

**Table 1:** Oligonucleotide sequences in this study.

Name	Sequence (5′ to 3′)
RPA forward primer	TACAGAGAAATTGGCCCAAGAGCCCCTTATAT
RPA reverse primer	GTTCTGATCAATTCCTCCAGCATGGTGACGTG
crRNA1	UAAUUUCUACUAAGUGUAGAUGCCUAGUCUGAAAUACAUAG
crRNA2	UAAUUUCUACUAAGUGUAGAUAGACUAGGCCAAAAAUCAGC^)^
crRNA3	UAAUUUCUACUAAGUGUAGAUGUCUGAAUUGAUUCUUCAAG
ssDNA reporter	FAM-TTATT-BHQ1
RT-qPCR forward primer	GGTATGARTGTTTTTTGTTTTGGTTTAC
RT-qPCR reverse primer	CGGCTTTTGYGAATTCTTGA
RT-qPCR Probe	ATCAAAACAGAGATGGGAGC

Abbreviations: BHQ, black hole quencher; FAM, carboxyfluorescein; RPA, recombinase polymerase amplification; RT-qPCR, reverse transcription quantitative polymerase chain reaction.

## Data Availability

The data will be made available upon request.

## Supporting Information

Supporting Information Figure S1: Detailed sequence information of the crRNAs targeting the NiV N gene. (a) Three crRNAs were designed on the basis of the position of the protospacer adjacent motif (PAM) in the NiV N gene. (b) Sequence conservation of three crRNAs in 112 aligned NiV strains. Figure S2: Feasibility of the one-pot visual RT-RPA-CRISPR/Cas12 assay under room temperature conditions. (a) Schematic illustration of the one-pot assay under room temperature conditions. The EP tubes were sealed and placed in a thermal cycler or water bath at 25°C for 20 min to achieve amplification of the targets. The EP tubes were then instantaneously centrifuged to allow the CRISPR/Cas12a reaction system to mix with the RT-RPA reaction components, and the samples were reacted at 25°C for 40 min. The results were observed via a mobile phone or with the naked eye under a blue light meter (Major Science Shanghai). (b) The detection limit of the assay for NiV-positive plasmids at room temperature. (c) The detection limitation of the assay for NiV-positive plasmids in a nonlaboratory setting (29–30°C). NTC, nontarget control reaction. The values represent the means ± s.d. from three replicates (ordinary one-way ANOVA; *⁣*^*∗∗*^*P* ≤ 0.01; *⁣*^*∗∗∗*^*P* ≤ 0.001).
