# Supplementary material for: A Single-Copy Sensitive and Field-Deployable One-Pot RT-RPA CRISPR/Cas12a Assay for the Specific Visual Detection of the Nipah Virus
Source: Transbound Emerg Dis. 2024 Nov 20;2024:4118007. doi: 10.1155/2024/4118007 (PMC12017183; doi:10.1155/2024/4118007)
Supplement: Supporting Information — Figure S1: Detailed sequence information of the crRNAs targeting the NiV N gene. (a) Three crRNAs were designed on the basis of the position of the protospacer adjacent motif (PAM) in the NiV N gene. (b) Sequence conservation of three crRNAs in 112 aligned NiV strains. Figure S2: Feasibility of the one-pot visual RT-RPA-CRISPR/Cas12 assay under room temperature conditions. (a) Schematic illustration of the one-pot assay under room temperature conditions. The EP tubes were sealed and placed in a thermal cycler or water bath at 25°C for 20 min to achieve amplification of the targets. The EP tubes were then instantaneously centrifuged to allow the CRISPR/Cas12a reaction system to mix with the RT-RPA reaction components, and the samples were reacted at 25°C for 40 min. The results were observed via a mobile phone or with the naked eye under a blue light meter (Major Science Shanghai). (b) The detection limit of the assay for NiV-positive plasmids at room temperature. (c) The detection limitation of the assay for NiV-positive plasmids in a nonlaboratory setting (29–30°C). NTC, nontarget control reaction. The values represent the means ± s.d. from three replicates (ordinary one-way ANOVA; ⁣∗∗P ≤ 0.01; ⁣∗∗∗P ≤ 0.001). [file 4118007.f1.docx]

Supplementary Information for

**A single-copy sensitive and field-deployable one-pot RT-RPA CRISPR/Cas12a assay for the specific visual detection of the Nipah virus**

**Supplemental Figures**

**
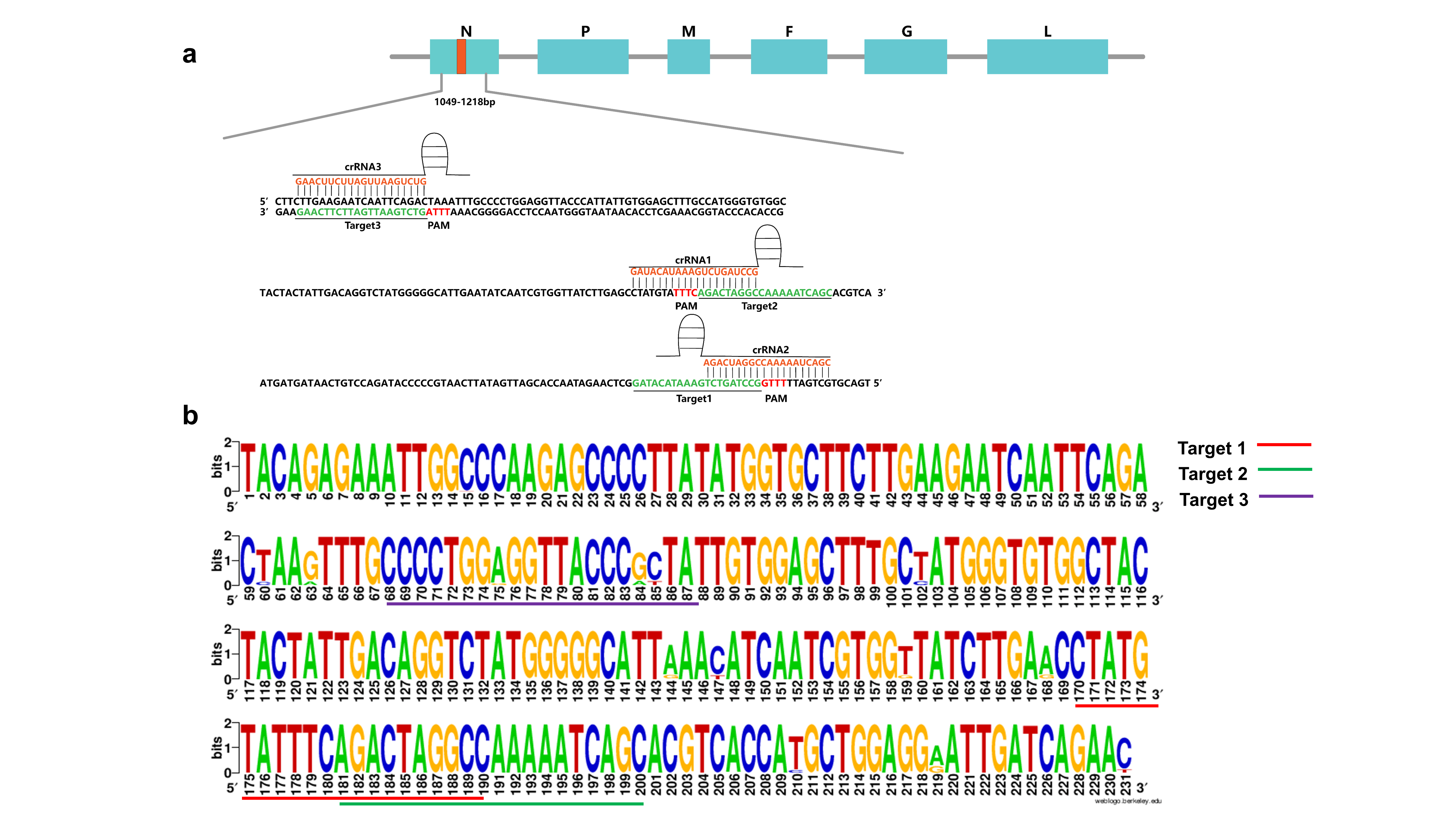
**

Figure S1: Detailed sequence information of the crRNAs targeting the NiV N gene. (a) Three crRNAs were designed on the basis of the position of the protospacer adjacent motif (PAM) in the NiV N gene. (b) Sequence conservation of three crRNAs in 112 aligned NiV strains.


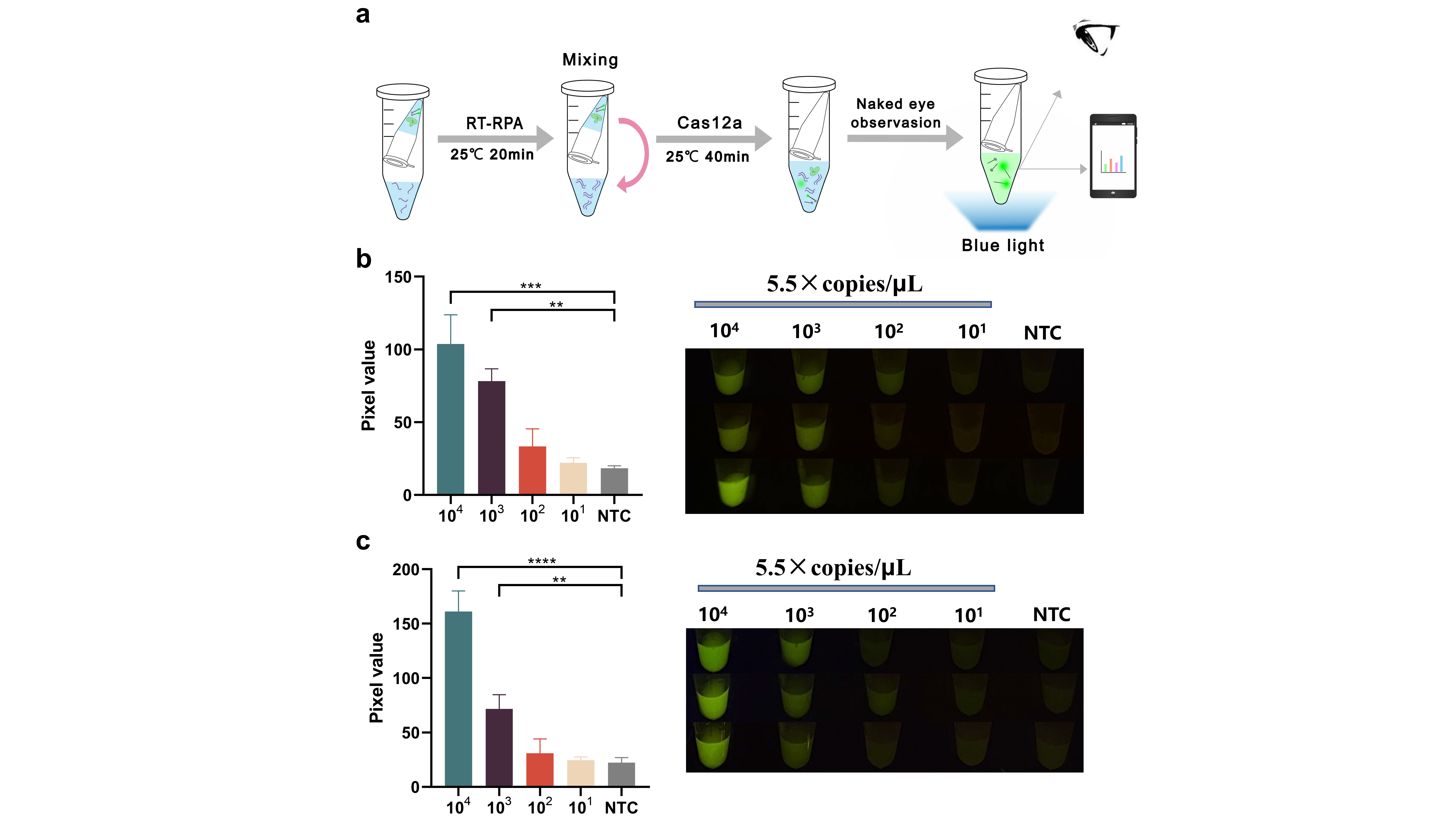


Figure S2: Feasibility of the one-pot visual RT-RPA-CRISPR/Cas12 assay under room temperature conditions. (a) Schematic illustration of the one-pot assay under room temperature conditions. The EP tubes were sealed and placed in a thermal cycler or water bath at 25°C for 20 min to achieve amplification of the targets. The EP tubes were then instantaneously centrifuged to allow the CRISPR/Cas12a reaction system to mix with the RT-RPA reaction components, and the samples were reacted at 25°C for 40 min. The results were observed via a mobile phone or with the naked eye under a blue light meter (Major Science Shanghai). (b) The detection limit of the assay for NiV-positive plasmids at room temperature. (c) The detection limitation of the assay for NiV-positive plasmids in a nonlaboratory setting (29-30°C). NTC, non-target control reaction. The values represent the means ± s.d. from three replicates (Ordinary one-way ANOVA; ** P ≤ 0.01; *** P ≤ 0.001).
